# Supplementary material for: Linear and categorical coding units in the mouse gustatory cortex drive population dynamics and behavior in taste decision-making
Source: bioRxiv. 2025 Oct 7:2025.10.06.680705. Preprint. [Version 1] doi: 10.1101/2025.10.06.680705 (PMC12632523; doi:10.1101/2025.10.06.680705)
Supplement: Supplement 1 [file NIHPP2025.10.06.680705v1-supplement-1.pdf]

bioRxiv preprint doi: https://doi.org/10.1101/2025.10.06.680705; this version posted October 7, 2025. The copyright holder for this preprint (which was not certified by peer review) is the author/funder, who has granted bioRxiv a license to display the preprint in perpetuity. It is made available under aCC-BY 4.0 International license.

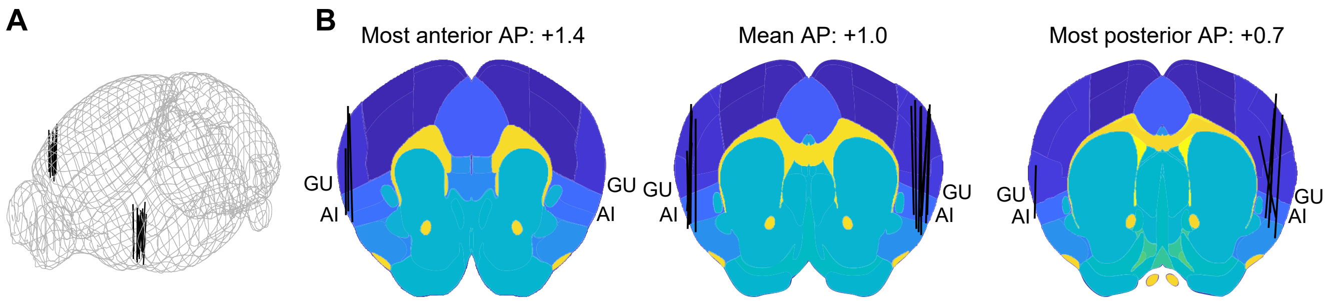


**Supplementary Figure 1. Neuropixels probe trajectory reconstruction. A**: 3D reconstruction of the 23 probe trajectories from the experimental dataset. **B**: 2D reconstruction of the same 23 probe trajectories, overlaid on the Allen Brain Atlas at varying anteroposterior (AP) distances (relative to Bregma in mm) around GC. At these coordinates, both GU (gustatory areas) and AI (anterior insular areas) account for GC. Reconstructions performed with open-source Allen CCF Tools (***Shamash et al., 2018***; github.com/cortex-lab/allenCCF).

bioRxiv preprint doi: https://doi.org/10.1101/2025.10.06.680705; this version posted October 7, 2025. The copyright holder for this preprint (which was not certified by peer review) is the author/funder, who has granted bioRxiv a license to display the preprint in perpetuity. It is made available under aCC-BY 4.0 International license.

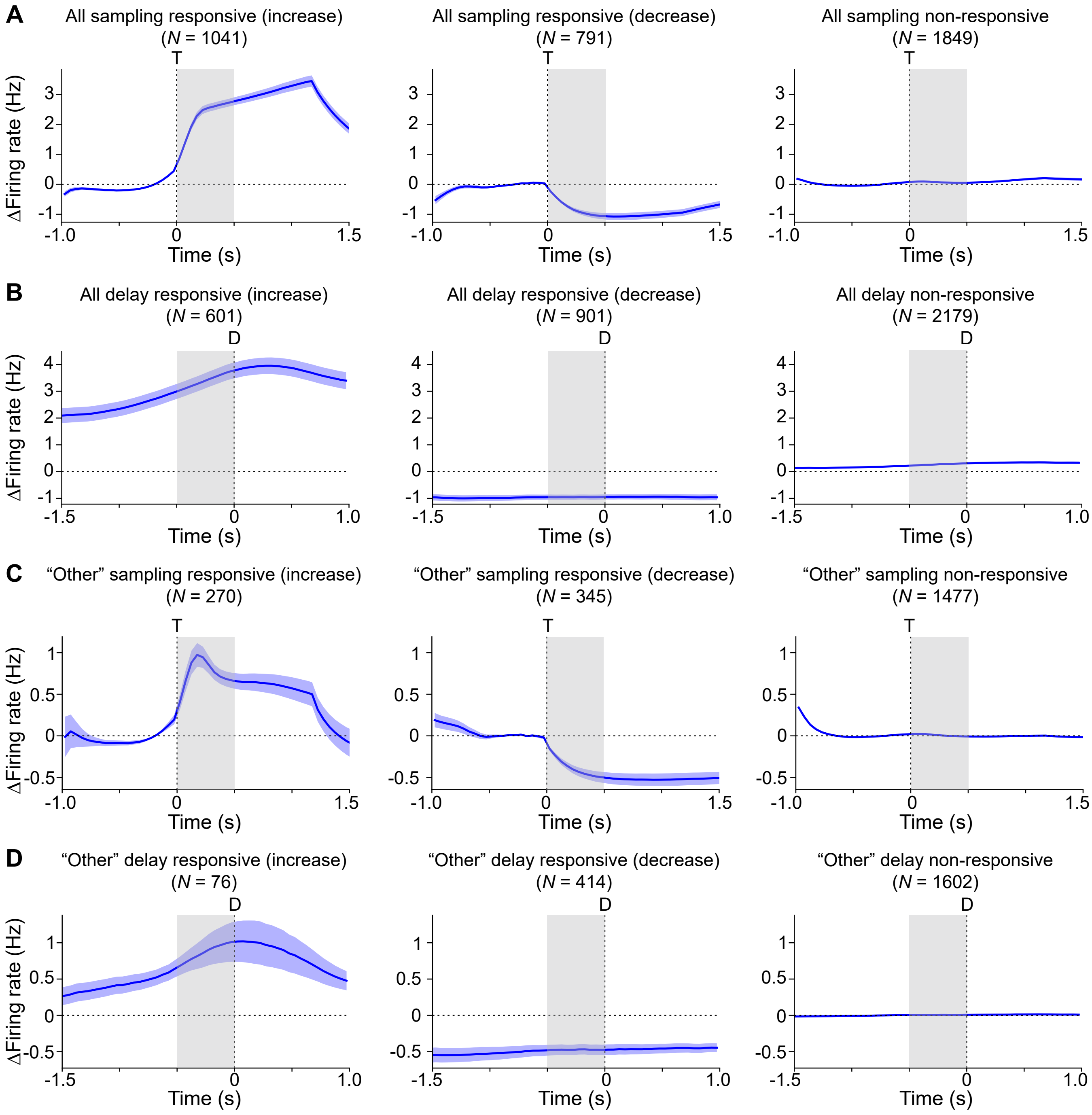


**Supplementary Figure 2. RNN unit responsiveness. A**: Activity of all RNN units grouped by responsiveness during the sampling period. If the unit's firing rate distribution during the sampling period (T to T + 0.5 s) was significantly different from its baseline (T – 0.5 s to T) firing rate distribution, it was sampling responsive and grouped by whether its mean firing rate increased (left) or decreased (middle); otherwise it was non-responsive (right). **B**: Activity of all RNN units grouped by responsiveness during the delay period. Same as **A** except the firing rate distribution of interest is calculated over D – 0.5 s to D. **C** and **D**: Same as **A** and **B**, respectively, except that the only units considered are those labeled “other” by the response profile analysis of **Figure 6C**. Firing rates are expressed relative to baseline, and traces are population mean ± s.e.m.

bioRxiv preprint doi: https://doi.org/10.1101/2025.10.06.680705; this version posted October 7, 2025. The copyright holder for this preprint (which was not certified by peer review) is the author/funder, who has granted bioRxiv a license to display the preprint in perpetuity. It is made available under aCC-BY 4.0 International license.

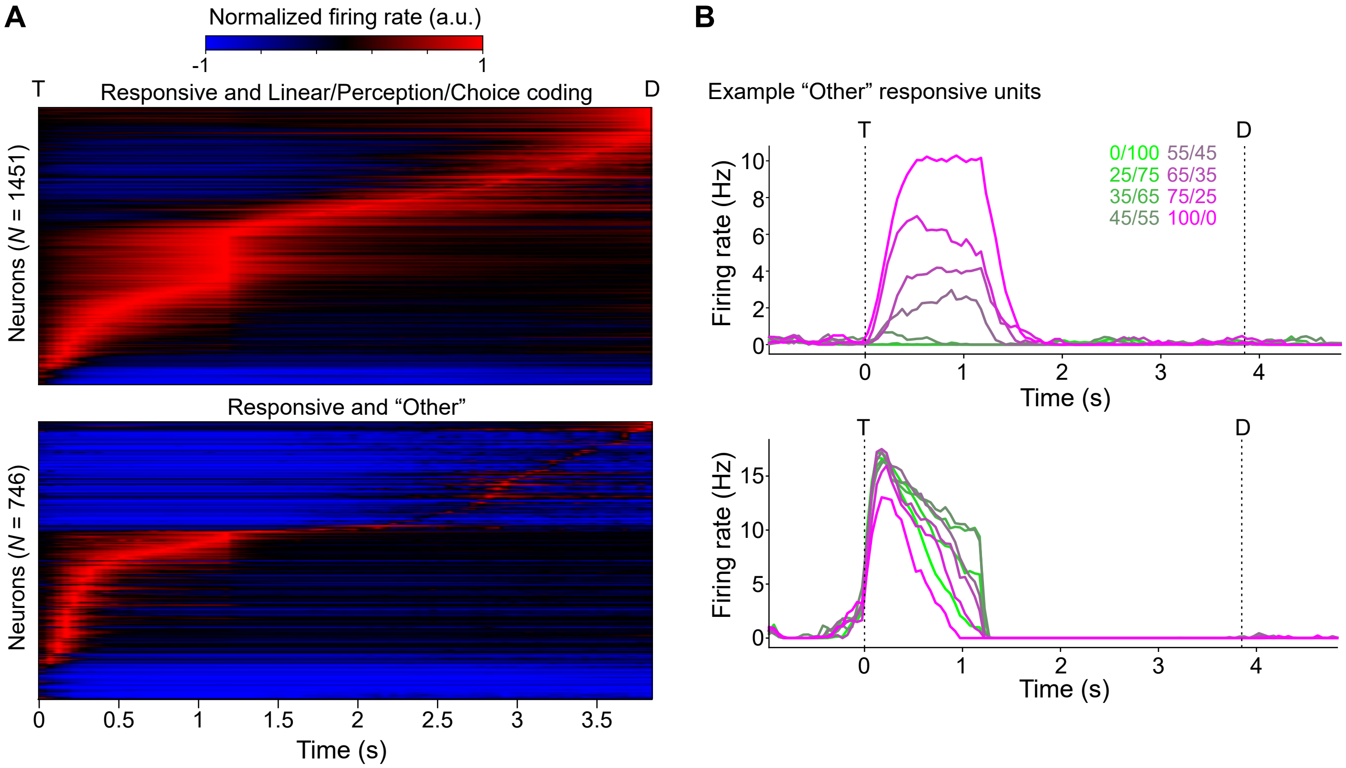


**Supplementary Figure 3. RNN unit activity patterns. A**: Heatmaps of firing rate activities for units that responded significantly during the sampling and/or delay periods, broken down into coding units (linear, step-perception, and/or step-choice) (top) and "other" units (not linear, not step-perception, and not step-choice) (bottom). Firing rates are expressed relative to baseline and normalized to the maximum absolute value. **B**: Two example "other" unit responses. Both respond significantly during the sampling period, but neither response pattern matches the linear or step templates. Color scale corresponds to different mixture stimuli (%Sucrose/%NaCl).
